# Photonic Programmable Tele-Cloning Network

**DOI:** 10.1038/srep28903

**Published:** 2016-06-29

**Authors:** Wei Li, Ming-Cheng Chen

**Affiliations:** 1 Department of Physics, Tongji University, Shanghai, P. R. China; 2 CAS-Alibaba Quantum Computing Laboratory, Shanghai, P. R. China

## Abstract

The concept of quantum teleportation allows an unknown quantum states to be broadcasted and processed in a distributed quantum network. The quantum information injected into the network can be diluted to distant multi-copies by quantum cloning and processed by arbitrary quantum logic gates which were programed in advance in the network quantum state. A quantum network combines simultaneously these fundamental quantum functions could lead to new intriguing applications. Here we propose a photonic programmable telecloning network based on a four-photon interferometer. The photonic network serves as quantum gate, quantum cloning and quantum teleportation and features experimental advantage of high brightness by photon recycling.

Quantum network^1^,^2^,^3^, a distributed quantum entangled state^4^,^5^, can spread and process the quantum information from external quantum state by injecting into its local network nodes. Then the post-processed quantum information could be extracted from the distant nodes of the quantum network. This paradigm, derived from the seminal concept of quantum teleportation^6^,^7^,^8^, has been developed into many novel quantum protocols, ranging from complex quantum state teleportation^9^,^10^, telecloning^11^,^12^, programmable quantum gates^13^,^14^, quantum gates using teleportation^15^ to one-way quantum computation^16^,^17^,^18^.

The cloning, transporting and processing are three basic information operations. An optimal quantum cloning machine^19^,^20^,^21^ is built to dilute N copies of initial identical quantum bits into M identical output copies with a maximal fidelity of 1 − (M − N)/(MN + 2M), which does not violate the quantum no-cloning theorem^22^. The quantum cloning machine^23^,^24^ can be implemented by a quantum network in the style of quantum teleportation, called quantum telecloning^11^, thus the quantum information copies are distributed among distant network nodes. In addition to the spread of quantum information, the network can also be programmed^13^ as a quantum computer^25^ to process the information simultaneously.

In this work, we propose a photonic quantum network of dual capacities for conveying and processing quantum information simultaneously – a photonic programmable telecloning machine. One copy of the quantum information was infused with the photon network through a Bell-state measurement. The information is further operated by a desired quantum gate programed in the entangled state of network. Two distant nodes are designed to output two copies of the post-processed information independently at the optimal quantum-cloning performance. In our photonic quantum network, four entangled photons are produced by the double emission of spontaneous parametric down-conversion (SPDC) in nonlinear crystal^26^. A photon recycling technology is used to carry the external quantum information, which will remarkably improve the efficiency of experimental realization.

## Theory

**Figure 1:**
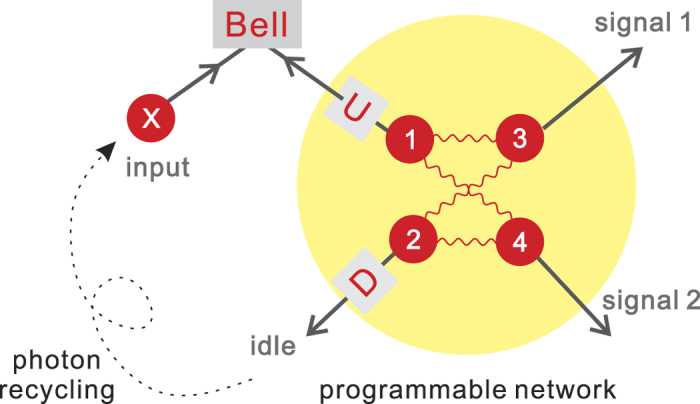
Programmable telecloning network. (**a**) Photons 1~4 are entangled in quantum state |ψ_*net*_〉 for telecloning and photon 1 is passed through a quantum gate *U* to program the desired quantum gate into the network state. (**b**) The operation of quantum network. The ports 3 and 4 are designed as the output ports. The photon in port 2 is depolarized before recycling to serve as the carrier photon X of external quantum information and further fused with the photon from port 1 through Bell measurement. Thus the carried quantum information is injected into the network through port 1 and the post-processed quantum information can be extracted from ports 3 and 4.

The programmable telecloning network^11^,^14^ is prepared in the four-party entangled state (up to normalization for simplicity) |*ψ*_*net*_〉_1234_ = 2|0000〉 + |0101〉 + |0110〉 + |1001〉 + |1010〉 + 2|1111〉 and is transformed by a quantum gate *U* on the port 1. Thus the resulted network state shown in Fig. 1 has the ability to spread and process quantum information. The photon 2 is depolarized and traced out from the network, which resulted in a mixed-state network 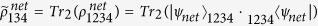. The input quantum information is carried by the recycled and reinitialized photon from port 2 with quantum state 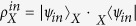, where the new photonic mode is tagged as X and 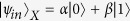.

To inject the quantum information from photon X into the network, we fuse it with the port 1 by a Bell-state measurement onto a quantum state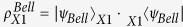 with 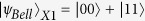. After the success of injection, the remained network state is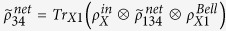. We find that the output ports 3 and 4 have quantum state 
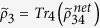
 and 
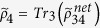
, which both are of quantum density matrix
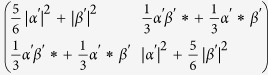
 with (*α*′ *β*′)^*T*^ = *U*(*α β*)^*T*^. These cloned quantum states are of fidelity 5/6 with programmable-gate-processed quantum state *U*(*α β*)^*T*^.

When the result of Bell measurement for the injection process is not |00〉 + 11〉, but other three Bell states, the quantum information in the network will be *Uσ*_*k*_(*α β*)^*T*^, where *σ*_*k*_ is one of the Pauli operators {*σ*_*X*_, *σ*_*Y*_, *σ*_*Z*_}. If the quantum gate *U* does not commutate with the Pauli operators, the Pauli errors presented between the quantum gate *U* and quantum state (*α β*)^*T*^ cannot be corrected by some universal method. In this sense, the programmable gate features a probabilistic nature with success probability of 1/4 and thus the partial Bell-state measurement realized by using linear quantum optics is not more a bottleneck.

### The proposed photonic network

**Figure 2:**
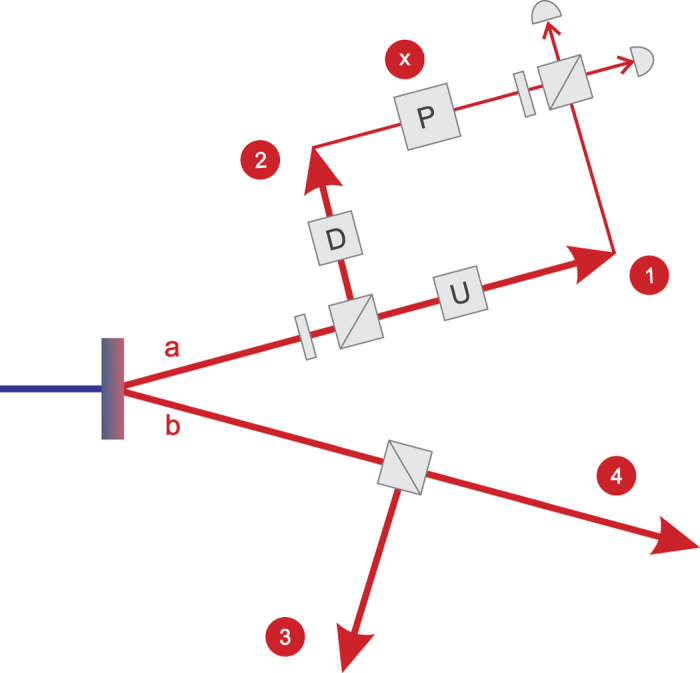
A nonlinear crystal pumped by a UV pulsed laser produces double IR entangled photon pairs by SPDC process. The two photon pairs further interfere with each other in the same paths. Two 50:50 beam splitters are used to separate the four photons into four different ports. The photons 1~4 constitute the programmable telecloning network, where the photon 1 is operated by a quantum gate *U* to program the desired gate into the state of network. The photon 1 played as the input port while the photons 3 and 4 served as two output ports. An external information carrier photon X will inject its quantum information into the input port by fusing with photon 1 through Bell measurement. To improve the network efficiency, the photon X is produced by depolarizing and recycling the idle photon 2.

We propose a photonic interferometer^27^ shown in Fig. 2 to realize the programmable telecloning network, where the horizontal (H) and vertical (V) polarization are used to encode the quantum state |0〉 and |1〉, respectively. The photons are produced by type-II SPDC^26^ and double emission is exploited to engineer the telecloning network. An additional half wave plate reverse the state in one SPDC mode, thus the initial photons state (up to normalization) is 
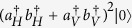
. According to the nature of bosons, when two identical photons present in a same mode, the probability amplitude will be enhanced by a factor 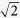, the quantum state of four photons is 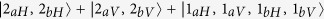. Two following beam splitters route the four photons to four separate ports 1~4 with a success probability 1/4, resulting in a four-party entangled state |*ψ*_net_〉_1234_, that is
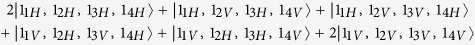


This photonic state will serve as the quantum network for pure telecloning. Furthermore, a quantum gate *U* is programmed in the network state by sending the photon in port 1 through wave plates with a quantum operation *U*. Now, the network state *U|ψ*_net_〉_1234_ , as programmable telecloning machine, is ready to spread and process quantum information.

In general, the external quantum information carrier will be a heralded single photon from a third photon pair of the SPDC emission. However, the SPDC process is probabilistic and the statistics of photon pairs follow the Poisson distribution. A trade-off between the brightness and the visibility of photon interference must be considered. An emission probability of a few percent is extensively used in quantum optical experiments^28^,^29^. Here, in our proposal, the photon in the idle port 2 of the network is exploited to serve as the carrier. Before the photon 2 is recycled, its information is erased through a depolarized channel. This can be viewed as we construct a mixed-state programmable telecloning network only with three ports 1, 3 and 4 (one as input port and two as output port).

The recycling photon, labelled as photon X, can be initialized to encode a quantum bit. Then it is send to the input port and joined with the photon 1 by Bell measurement. After a success detection of |1_*XH*_, 1_1*H*_〉 + |1_*XV*_, 1_1*V*_〉, the quantum bit in the photon X is injected into the network and concurrently processed by the same network. Now, the two photons in the output port 3 and 4 are two copies of the post-processed cloning state of optimal fidelity 5/6 with the post-processed prefect state.

In this sense, our photonic programmable telecloning network combines three foundational quantum information processing modules: programmable quantum gate, quantum cloning and quantum teleportation. There are four main aspects cause the system working in a probabilistic nature. First, the double emission of SPDC has a probability *p*^2^. Second, using two beam splitters to prepare the four-port entangled network state has an efficiency of 1/4. Third, the desired Bell measurement event on one beam splitter is of average 1/4 efficiency. Last, the efficiency of coupling, propagation loss and single-photon detections.

The probability of stochastic emission in SPDC is dominant factor for the low system efficiency. To improve the system efficiency, more pumping power can be used to increase the emission probability p. However, the noise contributed by the undesired high-order term emission will also increase. Thus a reasonable choice of the emission probability p is important. Here we simulate the system performance by random sampling 200 quantum state in the qubit Bloch sphere and investigate how the fidelity of telecloning change with the emission probability p, where the quantum gate U is set as identity gate I.

**Figure 3:**
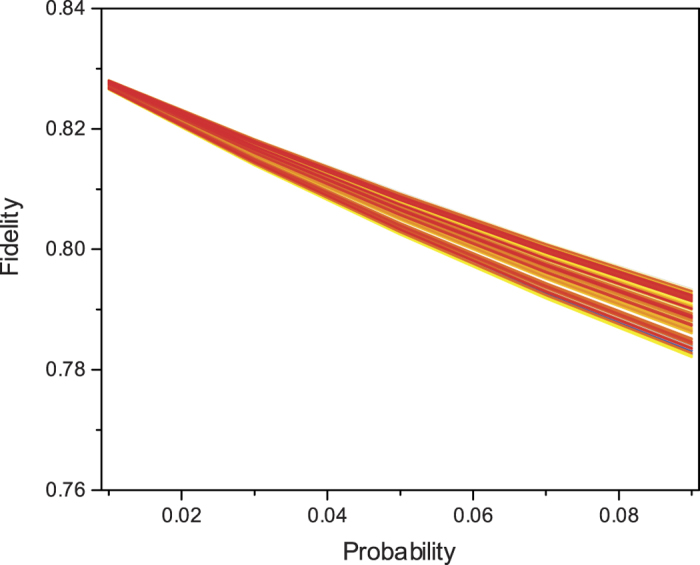
The performance of telecloning network. 200 random and uniform sampling quantum states, which are dense distributed in the Bloch sphere, are simulated. With the increasing of emission probability in SPDC, the average fidelity of the cloning states drops. In a broad region of emission probability, the working fidelities are all above the classical limit 2/3, showing the quantum nature of the network.

The simulation is performed in multi-mode phase space^30^. The photonic source is two-mode squeezed vacuum state and the single-photon detector only distinguish zero and non-zero photon numbers. Figure 3 shows the simulation results. We find that under small emission probability p, the average fidelity approaches to the theoretical vale 0.83. Then the fidelity dropped as the emission probability raising. In broad region of emission probability (from 0.01 to 0.1), all the telecloning fidelity is above the classical limit 0.66. Thus, to demonstrate the quantum nature of the telecloning network, we can appropriately use a high emission probability. However, to achieve the optimal cloning fidelity, low emission probability is essential.

## Conclusion

We have presented a new photonic quantum network with three basic quantum functions in quantum information processing. A programmable quantum computing on the diluted input information by quantum cloning in a distributed pattern on a versatile quantum network may find interest not only in the controlled distribution of quantum information, but also the inspiration of secure quantum computing.

## Additional Information

**How to cite this article**: Li, W. and Chen, M.-C. Photonic Programmable Tele-Cloning Network. *Sci. Rep.* **6**, 28903; doi: 10.1038/srep28903 (2016).
